# Solar thermotherapy reduces the titer of *Candidatus* Liberibacter asiaticus and enhances canopy growth by altering gene expression profiles in HLB-affected citrus plants

**DOI:** 10.1038/hortres.2017.54

**Published:** 2017-09-27

**Authors:** Melissa M Doud, Yungsheng Wang, Michelle T Hoffman, Christina L Latza, Weiqi Luo, Cheryl M Armstrong, Tim R Gottwald, Liangying Dai, Feng Luo, Yongping Duan

**Affiliations:** 1 United States Department of Agriculture-Agriculture Research Service-United States Horticultural Research Laboratory, Fort Pierce, FL 34945, USA; 2 College of Plant Protection, Hunan Agricultural University, Changsha 410128, China; 3 School of Computing, Clemson University, Clemson, SC 29634-0974, USA; 4 Center for Integrated Pest Management, North Carolina State University, Raleigh, NC 27606, USA

## Abstract

Huanglongbing (HLB), a systemic and destructive disease of citrus, is associated with ‘*Candidatus* Liberibacter asiaticus’ (Las) in the United States. Our earlier work has shown that Las bacteria were significantly reduced or eliminated when potted HLB-affected citrus were continuously exposed to high temperatures of 40 to 42 °C for a minimum of 48 h. To determine the feasibility and effectiveness of solar thermotherapy in the field, portable plastic enclosures were placed over commercial and residential citrus, exposing trees to high temperatures through solarization. Within 3–6 weeks after treatment, most trees responded with vigorous new growth. Las titer in new growth was greatly reduced for 18–36 months after treatment. Unlike with potted trees, exposure to high heat did not eradicate the Las population under field conditions. This may be attributed to reduced temperatures at night in the field compared to continuous high temperature exposure that can be maintained in growth chambers, and the failure to achieve therapeutic temperatures in the root zone. Despite the presence of Las in heat-treated commercial citrus, many trees produced abundant flush and grew vigorously for 2 to 3 years after treatment. Transcriptome analysis comparing healthy trees to HLB-affected citrus both before and after heat treatment demonstrated that post-treatment transcriptional expression patterns more closely resembled the expression patterns of healthy controls for most differentially expressed genes and that genes involved with plant-bacterium interactions are upregulated after heat treatment. Overall, these results indicate that solar thermotherapy can be an effective component of an integrated control strategy for citrus HLB.

## Introduction

Citrus industries around the world are facing a substantial problem: how to maintain a productive crop when dealing with the challenge of huanglongbing (HLB). This disease, which can affect all species and hybrids of *Citrus*,^1^ is caused by a fastidious, phloem-limited bacterium, *Candidatus* Liberibacter asiaticus’ (Las) in the United States.^2^ This bacterium is spread by the phloem-feeding Asian citrus psyllid, *Diaphorina citri* Kuwayama (ACP).^2^ Disease symptoms include: one or more yellow shoots within an otherwise healthy looking canopy, leaves with asymmetrical blotchy mottle, corky veins and lopsided fruit that drop prematurely and contain aborted seeds.^2^ As the disease progresses, early fruit drop increases, yield and fruit quality decrease, and the infected tree eventually dies.^2,3^

HLB is present in most of the citrus growing countries of the world. Within the United States, the disease was first detected in Florida in 2005^2^ and is now widespread throughout the state with an estimated disease incidence of >95% (Gottwald unpublished data). In 2012, it was detected in two commercial orchards in Texas^4^ and is now present in multiple locations including residences. HLB is also threatening California, the second largest citrus production state in the US.^5^ ACP populations have been established in California since 2008 with the first HLB tree detected in 2012 at a residence in Los Angeles County.^6^ HLB has since been detected in 46 additional residential trees in Los Angeles and Orange Counties (as of March 2017), all within a ~13-mile radius of the initial detection.

HLB in the US is currently attributed to Las.^2,7^ Las, like the other two HLB-associated Liberibacter species, remains uncultured. The inability to culture the bacterium severely limits many avenues of research into the physiology, molecular biology and biochemistry of the bacterium, and limits testing of disease control strategies to *in planta* studies thus increasing the difficulty in designing an effective control strategy against HLB. At this time, there is no method to cure HLB-affected citrus trees in the field. Current management practices focus on the reduction and/or eradication of the ACP vector to prevent new infections^2^ combined with cleaner nursery materials and roguing of diseased trees to reduce inoculum in the field. Although significant progress towards understanding this disease complex has been made in the past 10 years, solutions, especially short-term solutions are urgently needed to sustain the $9 billion dollar citrus industry in Florida,^8^ the $200 million dollar industry in Texas,^9^ and protect the world’s citrus crop from further decline.

Various methods have been investigated to rescue diseased citrus including antibiotics.^10–13^ Although promising compounds have been found that reduce Las titers within an infected tree, Environmental Protection Agency regulations have restricted their use at this time.^10,14,15^ Previous research indicated that heat therapy (also known as ‘thermotherapy’) could be used to control HLB caused by Las.^16–18^ Our previous experiments using a controlled growth chamber determined that Las populations were no longer viable when exposed to continuous high temperatures.^16^ In these experiments, Las-infected citrus in one-gallon containers were exposed for various durations to temperatures reaching 40, 42, and 45 °C with 50% relative humidity in a controlled growth chamber. It was determined that continuous thermal exposure to 40–42 °C for a minimum of 48 h was sufficient to significantly reduce Las titer or eliminate Las bacteria entirely in HLB-affected citrus seedlings. In addition, seedlings exposed to high heat produced healthy new flush and grew with increased vigor.^16^

In this study, we investigated the use of thermotherapy as a method to mitigate HLB in a field setting. To determine whether thermotherapy is a feasible and successful HLB control method in the field, residential and commercial citrus trees were exposed to high temperatures using portable greenhouses to enclose trees in a high temperature environment induced by heat from solar radiation. Las titer in the canopy was measured periodically using quantitative real-time PCR (qPCR), and tree growth monitored over time. Additionally, the transcriptional profile of trees both before and after thermotherapy was obtained.

## Materials and methods

### Thermotherapy on residential citrus trees

**Figure 1:**
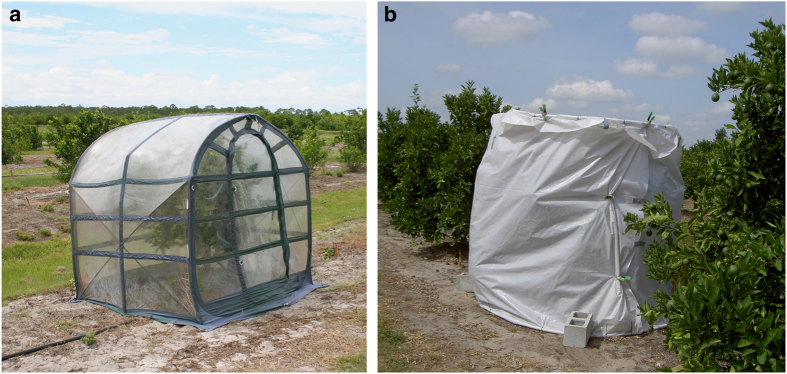
Thermotherapy enclosures. Enclosures consisted of commercially available SpringHouse Clear portable greenhouses 6′×6′×6.5′ manufactered by Flowerhouse, Clio, MI citrus (**a**) or tents made with PVC pipes and opaque polyethylene plastic sheeting, which are commonly used by a local citrus growers for treatment within commercial citrus orchards (**b**).

A residential orchard of Las-infected 12-year-old sour orange (*Citrus aurantium*) located in Fort Pierce, FL was used to monitor the effects of solar thermotherapy. The orchard contained two rows of trees spaced 12-feet apart with approximately 5-feet of space between trees in the same row. Trees were not hedged, irrigated, fertilized or treated with any pesticides before the experiment. A total of 26 trees were arbitrarily selected for thermotherapy between 16 August and 7 October 2011 before the weather turned cooler (Table 1). Six additional trees were arbitrarily chosen as untreated controls. Control and experimental trees were located in both rows and all visually showed HLB symptoms. For thermotherapy, trees were first trimmed to fit within a sealed portable clear polyethylene plastic greenhouse (6′×6′×6.5′ SpringHouse Clear; Flowerhouse, Clio, MI, USA) (Figure 1a). The citrus trees remained covered for 7 sunny days except for heat period 2 which, due to the stormy weather, trees were covered for 9 days (Table 1). The addition of the 2 days helped ensure that temperature thresholds were reached for the appropriate length of times since the lack of constant sunshine during stormy conditions prevented high temperatures from being achieved within the plastic greenhouse. Local weather data was recorded from weather station KFPR (FPR) in Fort Pierce, FL, USA while conditions within the portable greenhouses were monitored using a HOBO U23 Pro v2 data logger (Onset, Bourne, MA, USA) to record temperature and relative humidity. Two applications of 0.5 lbs of slow-release 13-4-9 with minor’s fertilizer (Harrell’s Professional Solutions, Lakeland, FL, USA) were spread by hand on both untreated and treated trees during the first year after thermotherapy.

### Thermotherapy on commercial citrus trees

Portable greenhouses composed of PVC pipes and opaque polyethylene plastic sheeting were constructed and placed over commercial citrus to subject them to high heat temperatures generated from solar radiation (Figure 1b). Twenty-six Las-infected 5-year-old Valencia trees (*Citrus sinensis* cv. Valencia on Swingle) located in commercial Orchard I in Fort Pierce, FL were exposed to high heat for 3 days in April 2011. In an effort to minimize tissue burn in trees and increase the rate that commercial growers could apply the therapy, a shortened duration was chosen and its effects on Las titer were determined. An additional 16 Las-infected 5-year-old Valencia trees located in commercial Orchard II underwent thermotherapy for 3–5 days (depending on the weather) in May 2012. Trees were selected based on having visual HLB symptoms and were scattered throughout the blocks. Trees were managed by standard commercial citriculture practices for fertilization and periodic insecticide sprays.

### Sample preparation and DNA extraction

For each plant, symptomatic leaf samples were collected before treatment to determine the initial PCR threshold cycle (Ct) values for Las. Plants were sampled periodically after the treatment to assess changes in Las titer. Specifically, three larger branches with multiple sub-branches were marked. Five to seven fully expanded, hardened leaves (symptomatic, if present) were sampled from each of these three marked branches per tree. If a marked branch died after heat treatment, samples were taken from the branch closest to the junction of the dead branch. Leaf tissue was either immediately processed or stored at 4 °C and processed within 2 weeks.

Total genomic DNA was extracted using a modified Qiagen DNeasy Plant mini kit protocol (Qiagen, Germantown, MD, USA). In the modified Qiagen DNeasy Plant mini kit protocol, ~200 mg of leaf midrib from multiple leaves were finely cut and placed in sterile 2 ml tubes with silicone-carbide shards, 2.3 mm chrome-steel beads and 800 μl of AP1 extraction buffer. Tissue was macerated using a Fast Prep-24 homogenizer (MP Biomedical, Solon, OH, USA) at speed 6.5 for 120 s. Eight microliters of RNase A was added to the homogenized tissue and incubated for 30 min in a 65 °C water bath. Samples continued to be processed using the standard published Qiagen protocol. Final DNA samples were eluted in 70 μl of sterile water. Quality and quantity of DNA was determined using the Nanodrop 2000 spectrophotometer (Thermo Scientific, Wilmington, DE, USA). DNA was stored at −20 °C until further use.

### PCR methods

All real-time PCR amplifications were performed with an Eppendorf Mastercycler realplex thermal cycler (Eppendorf, Hauppauge, NY, USA) using the Las specific 16S rDNA primers HLBasf and HLBr (5 μM) and TaqMan probe HLBp (2.5 μM),^19^ TaqMan Fast Universal PCR Master Mix (2×), No AmpErase UNG (Life Technologies, Foster City, CA, USA), and 100 ng of total genomic DNA in a total reaction volume of 15 μL. Amplification settings were as follows: a 95 °C initial denaturation step for 5 min followed by 40 cycles of 95 °C for 3 s then 60 °C for 30 s. All reactions were performed in duplicate using the ‘fast’ temperature mode. For data comparison across real-time plates, the minimum threshold was set to 100 with drift correction selected. The mean threshold cycle (Ct) was used to estimate Las titer (cells per 100 ng of total genomic DNA) based on the standard curve developed in the laboratory. For the standard curve, a portion of Las 16S rDNA was amplified with the OI1 and OI2C primers^20^ and cloned into the pCR Topo 2.1 vector (Life Technologies, Grand Island, NY, USA). Genomic DNA from healthy citrus was spiked with the plasmid and used to generate a standard curve, that is, log copy number (CN)=12.85–0.29Ct, *r*=0.996. Given that Las contains three copies of the 16S rRNA,^7^ copy number is divided by three to determine the number of Las cells per 100 ng of total genomic DNA.

### Statistical analysis

All qPCR Ct results below the detection threshold level of Las 16S rDNA were arbitrarily assigned the value of 40, indicating zero detection after 40 cycles. For comparison purposes, Ct values were converted into Las cells per 100 ng of total genomic DNA based on the generated qPCR standard curve and then log transformed for normality. The effect of heat treatment on Las titers at different sampling time points were first analyzed by one-way analysis of variance with repeated measures. Pairwise comparisons between groups were determined by two-sided paired *t*-test with Bonferroni correction. Since sampling was repeated in time, time was originally treated as a factor. However, preliminary analysis treating sampling time as a factor showed no significant effect and thus it was removed from the analysis. All statistical analyses were performed using R V3.2.1 software^21^ with the level of significance set at 0.05.

### RNA extraction and high-throughput sequencing

Potted 2 to 3-year-old healthy and Las-infected Valencia trees (*Citrus sinensis* cv. Valencia) were grown in the greenhouse at the USDA USHRL facility in Ft. Pierce, FL, USA with a 12 h photoperiod. Young, tender leaves collected both before heat treatment and ~1 month after heat treatment were immediately frozen in liquid nitrogen and labeled as follows: DHTi=leaves from three Las-infected plants prior to undergoing heat treatment, DHTa=leaves from the three Las-infected plants after heat treatment and CTL=leaves from two healthy plants that acted as controls and remained under greenhouse conditions at all times. Heat treatment of potted plants was performed under controlled conditions in a Conviron growth chamber (Controlled Environments, Inc, Pembina, North Dakota, USA) and consisted of 4 h of 40 °C per day for 3 days after which, plants were returned to greenhouse conditions. Use of these short heating periods more closely mimicked the conditions that trees in the field were subjected to during diurnal solar thermotherapy. Total RNA was extracted from whole leaves of each sample according to the RNeasy Plant Mini Kit standard protocol (Qiagen Inc., Valencia, CA, USA). The quantity and quality of RNA was evaluated using a Nanodrop ND-1000 spectrophotometer. A total of 20–30 μg of RNA from each sample was sent to BGI-Hong Kong (China) for RNA sequencing. RNA-Seq libraries were constructed following the Illumina protocol of mRNA-sequencing sample preparation (Illumina Inc., San Diego, CA, USA). The quality of each library was examined using a BioRad Experion (BioRad, Hercules, CA, USA) and the high-throughput sequencing was carried out by BGI using HiSeq2000 (Illumina, San Diego, CA, USA).

### RNA-Seq data processing and analyzing

First, the raw Illumina reads were cleaned by removing low-quality reads (Q20) and trimming adaptor sequences. Then, clean reads from each sample were mapped to the citrus reference genome and the read numbers for each gene were counted using STAR^22^ with a maximum intron 5000 bp (−alignIntronMax 5000). The *Citrus clementina*^23^ reference genome and corresponding annotation were downloaded from the Citrus Genome Database (http://www.citrusgenomedb.org/). Differentially expressed (DE) genes between each group were identified using the DESeq2 Bioconductor package.^24^ Raw counts of each gene were normalized to adjust for different sequencing depths across samples using DESeq2. After estimating the dispersion of each gene, DESeq2 identified differentially expressed genes between groups using an adjusted *P*-value (FDR) threshold of 0.01. The KEGG enrichment analysis was carried out using the clusterProfiler Bioconductor package^25^ with *Citrus clementina* as the reference (use cic as the organism) and an adjusted *P*-value threshold of 0.05. Differential gene expression was verified by reverse-transcription real-time PCR using SYBR green (Quanta Biosciences, Beverly, CA, USA) and cDNA constructed from oligo dT primers with all subsequent amplification steps using gene specific primers.

## Results

### Solar thermotherapy achieved with portable greenhouses

Portable greenhouses employed as thermotherapy enclosures (Figure 1) were successfully deployed in a field setting. With minor trimming, enclosures were placed over citrus trees, secured and remained in place for the duration of the treatment period. Regardless of the type of plastic used, tree branches occasionally punctured small holes in the material, which were easily patched and did not affect the ability of the enclosure to increase air temperatures above ambient. Both types of thermotherapy enclosures, when used during the hotter months in Florida (March to September), increased the air temperature within the enclosure and enabled exposure of Las-infected citrus to temperatures in excess of 40 °C.

**Figure 2:**
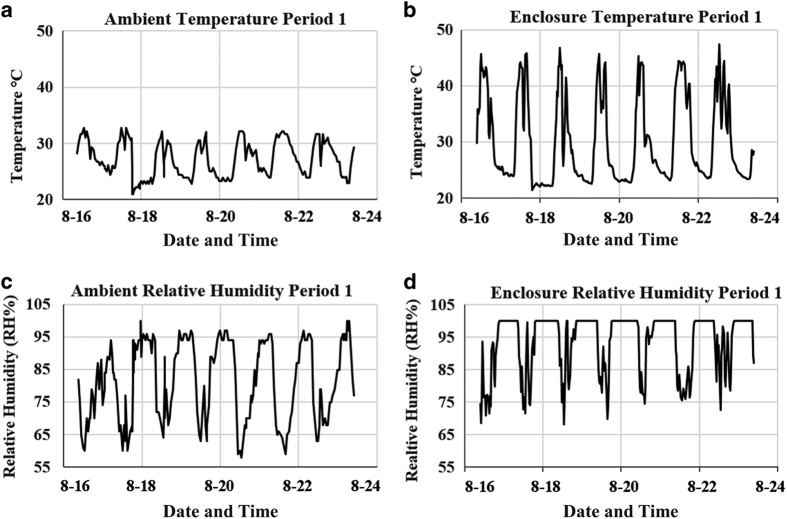
Thermotherapy enclosures increase temperature and relative humidity (RH) relative to ambient air. Ambient temperature (**a**) and RH (**c**) readings from the KFPR weather station located in Fort Pierce, FL were compared to temperature (**b**) and RH (**d**) data from heat period 1 recorded via a HOBO data logger placed on the center of the tree within a portable greenhouse located in a residential orchard. Data shown are for one tree during a treatment period but is representative of the data recorded within most enclosures.

Local weather data recorded from weather station KFPR (FPR) in Fort Pierce, FL, USA showed that ambient temperature and relative humidity (RH) readings varied throughout the day but never reached above 40 °C (Figures 2a and c, respectively). Typically, in Florida during the summer months, temperatures are lower in the morning, increase throughout the day and then quickly decrease in the afternoon usually due to cloud cover and daily rain storm events. After a storm, temperatures may gradually increase again until nightfall and are the lowest when it is dark. This daily weather pattern predominated during the period of August to October 2011 during the residential citrus experiments. Temperature readings were lowest before and after midnight with the highest peaks in the afternoon. During most days, the weather station recorded two temperature peaks in the afternoon, indicating a storm had brought cooler temperatures for a short period of time. Relative humidity also fluctuated and levels were quite high (58–100%) throughout the 24-hour period.

Temperature and relative humidity readings monitored inside the portable greenhouses used to treat residential citrus (Figure 1a) demonstrated that on most days, solar radiation raised the temperature within the portable greenhouse above 40 °C and simultaneously increased the relative humidity for a few hours (Figures 2b and d). Although ambient temperatures were high, cloudy days typically generated less heat within the enclosure. During all six treatment periods, temperatures above 40 °C were reached daily (3–8.5 h), with a maximum temperature of 56.8 °C. Relative humidity levels reached 100% daily and moisture was present on the side walls of the enclosures and on the leaves of the trees.

Temperature and relative humidity readings were also initially recorded in the commercial orchards to ensure that the alternatively constructed canopies, which were made from PVC pipes and opaque polyethylene plastic sheeting (Figure 1b), were also capable of producing thermotheraputic temperatures (data not shown). From this data, it was established that the alternatively constructed canopy when deployed in April and May could indeed produce internal temperatures above 40 °C, thus, additional enclosure readings were not recorded.

### Field thermotherapy reduces Las titer in infected trees

**Figure 3:**
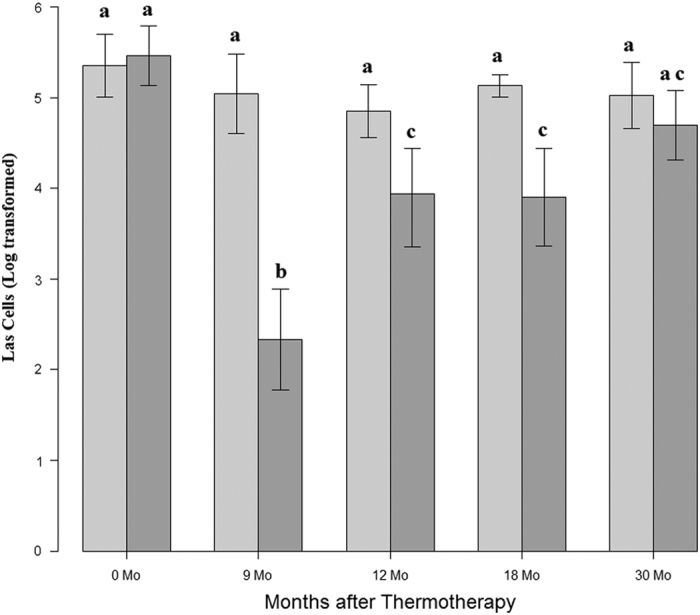
Reduction in Las titer post thermotherapy on residential trees. Las titers as defined as the log transformed Las cells/100 ng of genomic DNA were determined at time points 0, 9, 12, 18 and 30 months. Dark gray bars represent treated trees whereas light gray bars indicate control trees that did not undergo thermotherapy. Significance between time points is noted by different letters as determined by a two-sided paired *t*-test at a 95% confidence level. Error bars represent the standard error of the normalized average. Control *N*=6 and Treated *N*=26.

Las titers were tracked in both treated and non-treated trees. These data demonstrated that residential trees exposed to high temperatures showed a significant decrease in Las titers for 18 months post therapy compared to control trees (Figure 3; *P*<0.001). However, by 30 months, Las titers were no longer significantly different from the untreated control trees. The largest decrease in bacterial populations post thermotherapy was observed at 9 months, with an average of 9.84×10^3^ Las cells per 100 ng of genomic DNA in treated trees compared to the pre-treatment level of 6.37×10^5^ Las cells (Figure 3).

**Figure 4:**
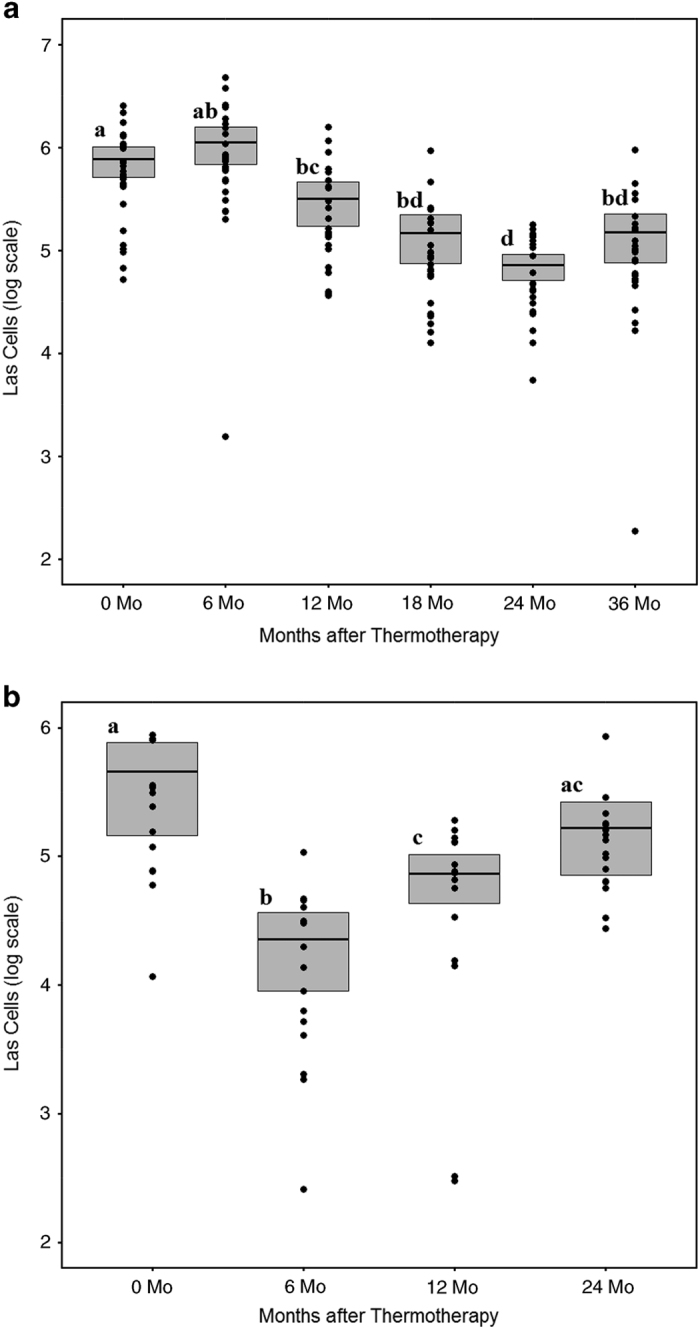
Effect of thermotherapy on Las titer in commercial orchards. Variations in Las titers for individual trees were plotted against time to determine the effectiveness of solar thermotherapy for commercial orchard I (**a**; *N*=26 trees) and commercial orchard II (**b**; *N*=16 trees). The mean is depicted as a black horizontal line with the standard error as the shaded area around the line. Las titer is defined as the log transformed Las cells/100 ng of genomic DNA. Significance between assessment times is noted by different letters as determined by a two-sided paired *t*-test at a 95% confidence level.

Trees within commercial orchards were also tested to determine the effectiveness of solar thermotherapy in a production setting. Variations in Las titers for individual trees treated with thermotherapy were monitored over time in two commercial orchards. In commercial orchard I, 26 trees had an average of 7.62×10^5^ Las cells per 100 ng of genomic DNA prior to treatment. At the six-month time point, most of the treated trees showed a decrease in Las titer. However, this decrease was not statistically significant because data from a few trees caused the overall average to be higher than the before treatment average. This increase may have resulted from sampling leaves that were present before thermotherapy. Our previous greenhouse study also showed that Las DNA is detectable after thermotherapy in older, hardened leaves that existed prior to the therapy although new leaves present after thermotherapy were negative for Las DNA.^16^ This phenomenon is thought to occur because the target DNA region appears to remain detectable via qPCR although the bacteria in the tissue is most likely dead.^26^ In addition, the hardened flush sampled at 12 months or later was collected from the terminal ends of the branch and are thus further away from tissue that existed before treatment; thereby, decreasing sampling error. Regardless, by 12 months the average Las titer (3.17×10^5^ Las cells per 100 ng of genomic DNA) was statistically lower than before treatment. This trend continued with significantly less bacteria at the 18, 24 and 36 months post treatment compared to the pretreatment levels (1.47×10^5^), (7.13×10^4^), and (1.50×10^5^), respectively; *P*<0.05) (Figure 4a). For commercial orchard II, which consisted of 16 trees, a decrease in Las titer was also seen after thermotherapy. Prior to treatment, an average of 4.58×10^5^ cells were detected per 100 ng of genomic DNA. At six and twelve months post treatment, the bacterial titer significantly decreased (2.28×10^4^) and (7.32×10^4^), respectively, (*P*<0.05) (Figure 4b).

### Tree growth and vigor increased as a result of heat treatment

**Figure 5:**
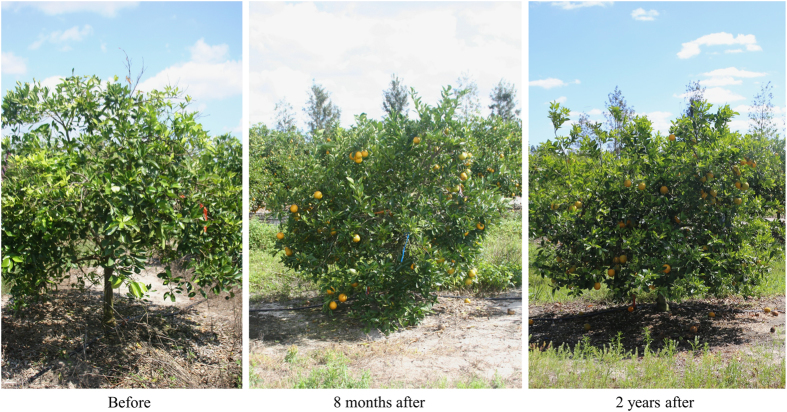
Phenotype of a commercial Valencia tree before and after thermotherapy treatment. This tree was exposed to high heat using the homemade portable greenhouse shown in Figure 1b in May 2012. Note yellowing and sparse canopy of the tree prior to treatment (before), whereas following treatment, abundant flush was produced by this tree for multiple years (8 months and 2 years after).

Observations on tree growth were noted before and after thermotherapy (Figure 5). Immediately after thermotherapy, leaves and small branches near the top of the tree occasionally displayed minor damage produced by the heat. However, unlike some other methods of thermotherapy, solar thermotherapy treatment did not result in substantial canopy defoliation and only a few leaves were lost from the treatment. Interestingly, all trees exposed to solar thermotherapy produced extensive new flush 3–4 weeks after the heat treatment, whereas, surrounding untreated trees did not flush during this same time period. Any visually symptomatic leaves prior to treatment remained symptomatic until the leaves naturally dropped over the following years. Canopy density was highly variable among trees. Many treated trees continued to generate asymptomatic flush and developed a larger, thicker canopy than was present before treatment for the following 2–3 years (Figure 5). However, a few treated trees declined in growth after the initial flush and displayed sparse canopies over the year following treatment. HLB symptoms were continuously present on leaves of untreated trees, which continued to decline. New foliage on trees post heat treatment was typically asymptomatic for the first few months but, thereafter, symptoms returned to some portion of the canopy.

### Transcriptional profiles revealed through RNA-Seq

In order to identify whether changes in gene expression were taking place in plants because of the thermotherapy treatment, the transcriptome was examined using RNA-seq both before treatment (DHTi) and one month after treatment (DHTa), with non-Las-infected plants used as controls (CTL). Greenhouse-maintained trees that were heat treated in a growth chamber were used for this analysis to help control for some of the variability associated with field-grown trees such as tree reinfection via ACP, weather associated temperature variations, and other biotic/abiotic stress factors such as herbivory and superinfection of the plants. In addition, this allowed a single source of inoculum to be used (something not possible with naturally infected field-grown trees) so that the effects seen from tree to tree would be more consistent considering the genetic diversity that has been shown to exist within Las.^27,28^ After cleaning the raw reads, >34.5 million reads were obtained for each sample (Table 2). Approximately 94.5% of the RNA-Seq reads mapped uniquely to the *Citrus clementina* ^23^ reference genome (Table 2), with reads mapping to 20,719 to 21,105 *C. clementina* genes, respectively.

### Differentially expressed genes related to Las infection and heat treatment

Initially, gene expression profiles were compared between healthy (CTL) and Las-infected (DHTi) citrus trees. From this, 5800 differentially expressed (DE) genes were identified using DESeq2 with an adjusted *P*-value <0.01 (Supplementary Table 1). Functional analysis showed that the 3118 upregulated genes were enriched in ribosome (*P*-adjust 2.27E−62) and ribosome biogenesis in eukaryotes (*P*-adjust 2.97E−16), and the 2683 downregulated genes were enriched in photosynthesis (*P*-adjust 3.5E−17) and photosynthesis—antenna proteins (*P*-adjust 3.2E−9).

Gene expression profiles were also compared between the DHTa and DHTi in order to identify expression changes that could be related to the heat treatment. A total of 1043 DE genes were detected between DHTa and DHTi using DESeq2 with adjusted *P*-value <0.01. Within these DE genes, 362 genes were significantly up-regulated and 681 genes were down-regulated in the heat-treated samples. These up-regulated genes were enriched in carbon metabolism (adjusted *P*-value 1.14E−5) and carbon fixation in photosynthetic organisms (adjusted *P*-value 1.46E−5), while the downregulated genes were enriched in ribosomal (adjusted *P*-value 2.99E−5) and ribosomal biogenesis genes in eukaryotes (adjust *P*-value 2.67E−25). Comparisons were also performed between the Las-infected group post heat-treatment (DHTa) and the healthy controls (CTL), with 2101 DE genes identified between these two groups (Supplementary Table 1).

### Expression profiles of post heat-treated infected-trees more closely resemble healthy trees

**Figure 6:**
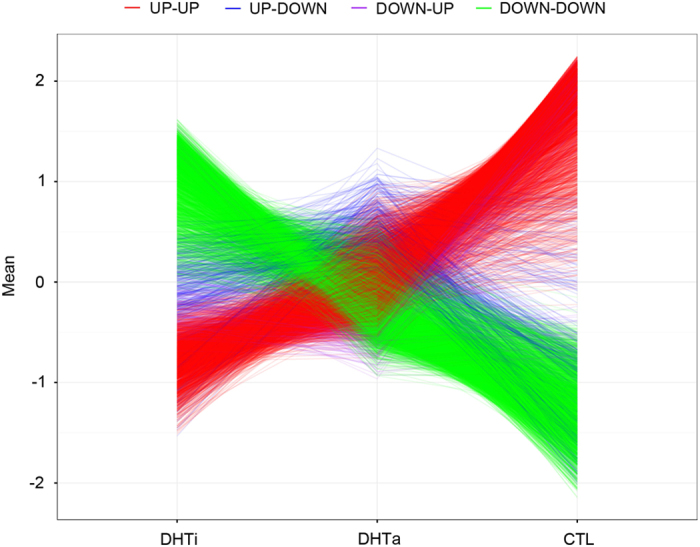
Expression patterns of 5800 DE genes between DHTi, DHTa, and healthy controls. Gene expression values were normalized using the *z*-score method with the mean expression values used to indicate the expression level of each gene in the group. DHTi denotes HLB affected citrus trees before heat treatment, DHTa denotes HLB affected citrus trees after heat treatment and CTL denotes healthy citrus trees. ‘UP-UP’ patterns indicate that gene expression was higher in DHTa compared to DHTi, and even higher in the control compared to DHTa. ‘DOWN-DOWN’ patterns indicate that gene expression was lower in DHTa compared to DHTi, and even lower in the control compared to DHTa. ‘UP-DOWN’ patterns indicate that gene expression was higher in DHTa compared to DHTi, but lower in the control compared to DHTa. ‘DOWN-UP’ patterns indicate that gene expression was lower in DHTa compared to DHTi, but higher in the control compared to DHTa.

A comparison of the gene expression patterns between DHTi, DHTa and CTL was performed (Figure 6). From this, it was noted that of the 2682 genes found to be downregulated in Las-infected plants compared to healthy controls, gene expression levels increase after heat treatment in 2520 of those genes. Furthermore, the gene expression levels of the 2851 genes upregulated in Las-infected plants (out of 3118) decreased after heat treatment. Remarkably, the overall gene expression pattern post heat treatment more closely resembled that of the healthy control, suggesting a correlation between specific transcriptional changes and disease recovery after heat treatment.

The 5800 DE genes in DHTi, DHTa, and the control groups could be further summarized by four expression patterns. If the gene expression was higher in DHTa compared to DHTi, and even higher in the control compared to DHTa, it was categorized as an ‘UP-UP’ pattern. If the gene expression was lower in DHTa compared to DHTi, and even lower in the control compared to DHTa, it was categorized as a ‘DOWN-DOWN’ pattern. If the gene expression was higher in DHTa compared to DHTi, but lower in the control compared to DHTa, it was categorized as an ‘UP-DOWN’ pattern. Finally, if the gene expression was lower in DHTa compared to DHTi, but higher in the control compared to DHTa, it was categorized as a ‘DOWN-UP’ pattern. As shown in Table 3, the most enriched KEGG pathways of the 2465 genes with the ‘UP-UP’ pattern were related to photosynthesis, which indicates that photosynthesis is more active after heat treatment than before. The most significant KEGG pathways of the 2824 genes with the ‘DOWN-DOWN’ pattern were those involving the ribosome, ribosome biogenesis in eukaryotes, the proteasome and the biosynthesis of amino acids. The reduced expression of ribosome-related genes seen here may be due to an overall reduction in Las after heat treatment. It was very interesting that the most enriched KEGG pathway for the 322 genes with the ‘UP-DOWN’ pattern involved plant-pathogen interactions.

### Candidate genes related to thermotherapy

In order to further understand the effect of heat treatment, we selected genes whose expression/repression was unique to heat treated samples. These genes were either significantly up-regulated or downregulated in DHTa compared to both DHTi and the control. We identified 4 genes (Ciclev10005101m, Ciclev10009440m, Ciclev1021471m and Ciclev10023753m) that were significantly up-regulated in DHTa compared to DHTi and the control (Supplementary Figure 1). Ciclev10005101m is a phosphoglycerate kinase (PGK), which is related to glycolysis and connected to the phenolpropanoid pathway. It was reported that PGK was activated in rice responding to *Rhizoctonia solani* Kuhn infection (Mutuku and Nose, 2012). Ciclev10021471m is annotated as BCL-2-associated athanogene 1 (BAG1). *BAG1* is involved in programmed cell death as well as regulation of stress in *Arabidopsis thaliana* plants^29^ and functions as a cofactor in Hsc70-mediated proteasomal degradation.^30^ Ciclev10023753m is a pseudo-response regulator 5 (*PRR5*) gene thought to be related to the circadian clock, which controls endogenous biological rhythms in eukaryotes. In *Arabidopsis*, *PRR5* plays an antagonistic role to CCA1, circadian clock associated 1.^31^ Ciclev10009440m belongs to the peroxisomal membrane 22 kDa (Mpv17/PMP22) protein family, whose function is currently unknown in plants. In *Saccharomyces cerevisiae*, SYM1, which shows homology to Mpv17, is induced by heat shock and is required for ethanol metabolism.^32^ We also identified 7 genes (Ciclev10014969m, Ciclev10010673m, Ciclev10004597m, Ciclev10001912m, Ciclev10020004m, Ciclev10004236m, Ciclev10011018m) that were significantly downregulated in DHTa compared to DHTi and the control (Supplementary Figure 2), although their function is mostly unknown in plants.

## Discussion

With citrus trees in Florida now in a severe stage of decline due to HLB, there is an urgent need for treatments that can be implemented immediately to eliminate or suppress Las and restore yields. Thermotherapy may meet these needs since it is environmentally friendly, does not require a regulatory permit, is suitable for both organic and conventional farming, and can be quickly adopted by growers. Since our prior study demonstrated that a continuous period of high heat can kill Las in potted trees and improve growth of the cured citrus tree,^16^ the present study was performed to define the effects of exposing Las-infected citrus in the field to high temperatures using portable greenhouses heated by solar radiation. Temperature readings showed that the conditions within the greenhouse/enclosures exceeded 40 °C, which were similar to the temperatures shown to be effective at suppressing Las in growth chamber studies.^16^ As expected, temperatures >40 °C were only maintained for short periods (3 to 8.5 h per day), with temperature range and duration within enclosures being variable. Thus, solar thermotherapy did not meet the cumulative number of hours for Las eradication therapy as determined in the controlled growth chamber study.^16^ Additionally, beneath the portable greenhouses only a slight change in soil temperature was detectable up to three inches below the surface with no change observed at a depth of eight inches below ground (data not shown). Despite the shorter exposure time, temperature conditions observed within the portable greenhouses did positively affect tree growth and suppressed Las titer 1 to 2 years post treatment.

Before treatment, control trees and the trees selected for thermotherapy at the residential location had similar levels of Las. Titers in the control trees remained similar throughout the sampling period while trees that were exposed to thermotherapy had significant changes in Las titer over time (Figure 3). Treated residential trees were shown to have significantly less Las bacteria than the control trees for 18 months after thermotherapy. However, the effect was not indefinite and Las bacterial levels began to rise between 12 and 30 months post treatment, although it was not until month 30 that the Las population reached levels equivalent to those in control trees. A similar pattern of initial decline in Las after thermotherapy with a subsequent increase in titer over time was also present in commercial orchard I and II (Figures 4a and b). The resurgence of titer levels over time is most likely due to the inability of solar thermotherapy to suppress Las in the root system. HLB is a systemic disease, which means that the Las bacteria is present in the phloem above ground as well as in the root system.^2^ Because solar thermotherapy did not generate enough heat to increase soil temperatures and the heat that was present under the enclosure may have not have been able to penetrate the bark on the trunk, Las cells colonizing the phloem in the root and trunk regions would have been unaffected by the heat. Over time, live bacteria could spread into the canopy again. Suboptimal duration times of solar thermotherapy and the emergence of heat-adapted bacterial subpopulations would also have the same effect. An increase in Las titers after thermotherapy due to reinfection by the psyllid vector is also likely. The ACP population was not controlled at the residential location and although insecticides were used in the commercial planting, ACP control is not 100% effective. The rate of Las titer increase varied between trees that would be indicative of incomplete ACP control and reinfection resulting in variable titer among trees. Future research is needed to investigate how factors e.g. rootstock, scion, size and age of the tree, duration of thermotherapy and temperatures generated influence the rate and level of change in Las titers.

Field thermotherapy stimulated growth in diseased trees. Photographic images showed that trees with advanced HLB have dieback, which causes the canopy to have a sparse appearance.^33^ All trees exposed to high heat produced a marked volume of flush within a month resulting in a visibly denser canopy (Figure 5). The appearance and amount of symptomatic leaves present after heat treatment was highly variable. Typically, leaves that developed from the first post-heat flush remained asymptomatic for at least 6 months. Despite a measurable level of Las in the leaves, some trees showed few symptoms of HLB until a year post treatment. Thus, the drop in bacterial titer resulting from the thermotherapy apparently slowed down the appearance of disease symptoms such as blotchy mottle, the classic symptom that is diagnostic of HLB and differentiates it from other diseases and maladies. The abundance of flush later post-treatment varied from tree to tree, with a portion of treated trees generating dense canopies for 2 years, whereas, another portion showed dieback after only 1 year post heat treatment. In citrus orchards affected by HLB, the trees not only acquire the infection at different times by different psyllids but they also decline at variable rates. Therefore, the tree to tree variability observed in response to treatment during this study could be due to the stage of infection/decline the individual trees were in at the time of treatment. Those further along in decline appear less likely to respond to solar thermotherapy, but more investigation is needed to verify these observations. Thus, ongoing, multi-year field trials are currently collecting data regarding the physical changes to trees produced by exposure to solar thermotherapy.

Genetic variation may play a role also in a tree’s ability to respond to thermotherapy. Given the 5800 genes found to be differentially regulated between the healthy and Las-infected citrus trees before treatment, HLB infection may be affecting a large portion of the citrus transcriptome. Large numbers of DE genes have also been reported in other studies comparing healthy to HLB-affected citrus^34–36^ with differences in number among studies possibly related to technique (RNA-Seq versus microarray), leaves sampled (young flush versus symptomatic tissue), and stage of infection. Based on the particular genes being differentially regulated, several interesting points can be inferred from the present study. For example, the upregulation in ribosome-related genes (Supplementary Table 1) seen in diseased samples before heat treatment compared to healthy samples implies that Las-infected citrus trees are able to translate more genes and make more proteins, thus consuming more energy. On the other hand, the simultaneous downregulation of photosynthesis-related genes in these plants reduces their energy supply. This unbalanced energy source-sink may cause the leaves to yellow,^37^ a typical symptom of HLB infected citrus trees. Previous research has also showed that HLB-infected citrus trees may have immune dysregulation and metabolic dysfunction caused by a source-sink disruption^38^ thus, our results are in agreement with these studies. Another implication centered around the differential regulation of a particular set of genes involves the fact that the most enriched KEGG pathway for the genes with the ‘UP-DOWN’ pattern (where gene expression is higher in DHTa compared to DHTi and the control) involved plant-pathogen interactions because it indicates that heat treatment may be activating resistance to HLB in citrus trees. This activation could originate from the release of the particles containing pathogen-associated molecular patterns upon the death of the Las cells. Irrespective of the mode of activation, the addition of heat appears to induce common regulatory pathways associated with responses to biotic as well as abiotic stress factors. Additional support for this can be found in a proteomics analysis where total protein extracts from heat-treated, Las-positive, *Citrus paradisi* trees were compared to healthy trees that had not undergone heat-treatment.^39^ Because several of the same genes were found to be upregulated in the heat-treated trees in both the current study (Supplementary Table 1) and the proteomics study by Nwugo *et al.*, especially those chaperones associated with heat shock, it can be concluded that a common response exists. In addition, consistent patterns of regulation were found in spite of the fact that the present study was performed on Valencia sweet orange trees and the proteomics analysis was performed on Duncan grapefruit, hence, strengthening the hypothesis that common stress pathways are induced in Las-infected citrus plants through heat regardless of citrus species.

Lastly, it is interesting to note that the number of genes differentially regulated between samples before treatment and healthy controls (5008) is significantly higher than the number between samples taken after treatment and the healthy control (2101), despite the fact that samples were initially infected with HLB. This, together with the fact that the overall gene expression pattern post heat treatment more closely resembled that of the healthy control, indicates that there may be a transcriptional signature associated with a healthy tree. The existence of such a signature could lead to rapid ways to access novel control methods being developed for HLB control.

In conclusion, although solar thermotherapy did not completely eradicate the Las population under field conditions, many trees did show vigorous growth for 2–3 years after treatment despite the presence of Las, indicating solar thermotherapy could be effective as part of an integrated control strategy for citrus HLB. In addition, transcriptome analysis demonstrated that the gene expression profiles of HLB-affected trees post heat-treatment more closely modeled healthy trees than their gene profiles prior to treatment, with many genes involved in plant-bacterium interactions being upregulated post treatment. How these gene expression patterns change over time in both treated and untreated trees and their correlation to HLB progression is a topic for future investigation.

## Figures and Tables

**Table 1:** Thermotherapy parameters for residential citrus

*Heat period (date and time)*^a^	*Duration (afternoons)*^b^	*No. of trees*
1 (08/16/11 09:30–08/23/11 09:30)	7	2
2 (08/30/11 08:00–09/08/11 11:00)	9	3
3 (09/08/11 13:00–09/15/11 09:30)	7	6
4 (09/15/11 10:00–09/22/11 10:00)	7	6
5 (09/22/11 11:00–09/30/11 09:00)	7	6
6 (09/30/11 12:00–10/07/11 11:00)	7	3
Control	0	6

a Portable greenhouses were sealed and placed over the tree for the entire time frame to expose trees to high heat. Control trees were not in an enclosure and only exposed to outside ambient temperatures.

b Duration is based on how many afternoons the trees were covered.

**Table 2:** RNA-Seq reads and mapping information

*Sample ID*	*Group*	*Total_reads number*	*Uniq_mapped_reads number*	*Mapped gene number*
Neg1	CTL	34,828,100	32933487	20731
Neg2		34,743,006	32861962	20768
DHTi1	DHTi	34,846,014	33238584	20942
DHTi2		34,818,108	33103221	21105
DHTi3		34,716,602	32958250	20910
DHTa1	DHTa	34,702,690	32855332	20719
DHTa2		34,542,751	32763906	20891
DHTa3		34,527,037	32731725	21164

**Table 3:** KEGG enrichment analysis of gene groups with different expression patterns

*Pattern notation*	*Gene expression pattern*	*Number of genes*	*Enriched KEGG pathway (adjusted* P*-value)*
UP–UP	DHTi<DHTa<CTL	2465	Photosynthesis (3.46E−17) Photosynthesis—antenna proteins (1E−9) Carbon fixation in photosynthetic organisms (2.76E−5)
DOWN–DOWN	DHTi>DHTa>CTL	2824	Ribosome (3.01E−67) Ribosome biogenesis in eukaryotes (2.05E−17) Proteasome (2.12E−7) Biosynthesis of amino acids (7.01E−7)
UP–DOWN	DHTi<DHTa>CTL	322	Plant-pathogen interaction (0.03)
DOWN–UP	DHTi>DHTa<CTL	189	Ribosome (0.0006)
