# Supplementary figures and images for: Solar thermotherapy reduces the titer of Candidatus Liberibacter asiaticus and enhances canopy growth by altering gene expression profiles in HLB-affected citrus plants
Source: Hortic Res. 2017 Sep 27;4:17054–. doi: 10.1038/hortres.2017.54 (PMC5615044; doi:10.1038/hortres.2017.54)

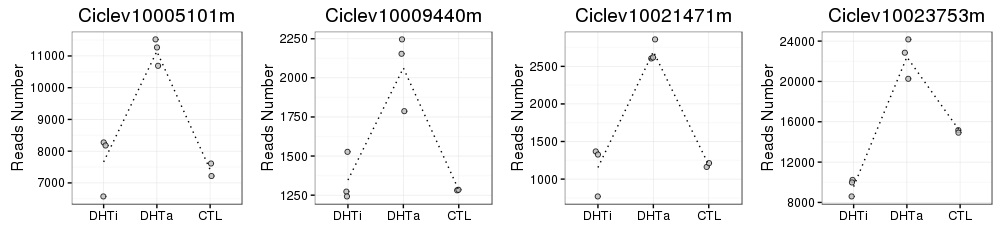

Supplement: Supplementary Figure 1 [file hortres201754-s2.jpg]

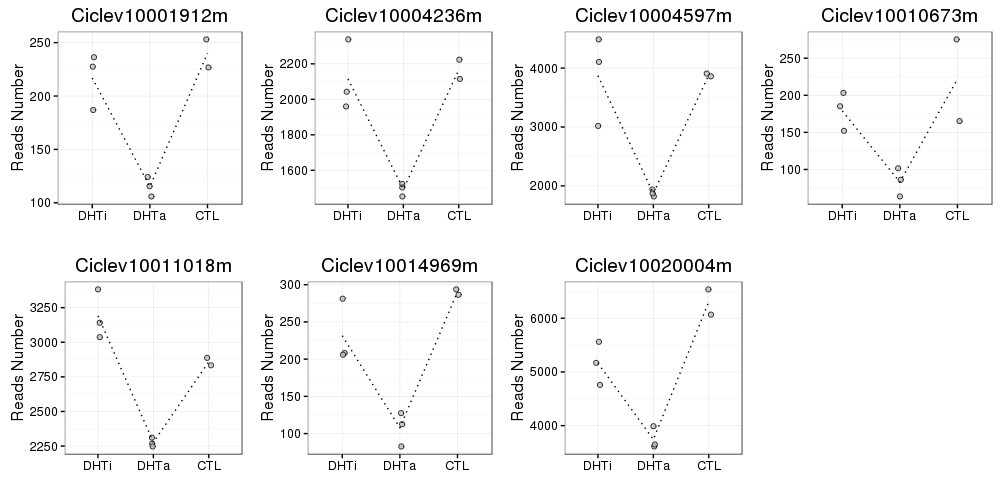

Supplement: Supplementary Figure 2 [file hortres201754-s3.jpg]
